# Ultrasonic cavitation induces necrosis and impairs growth in three-dimensional models of pancreatic ductal adenocarcinoma

**DOI:** 10.1371/journal.pone.0209094

**Published:** 2018-12-31

**Authors:** Einas Abou Ali, Benoit Bordacahar, Jean-Louis Mestas, Frederic Batteux, Cyril Lafon, Marine Camus, Frederic Prat

**Affiliations:** 1 Cochin Hospital, Gastroenterology and Endoscopy Department, Paris, France; 2 Cochin Institute, Paris, France; 3 Inserm, U1032, LabTau, Lyon, France; Université de Lyon, Lyon, France; 4 Paris Descartes University, Paris, France; University of South Alabama Mitchell Cancer Institute, UNITED STATES

## Abstract

**Introduction:**

Pancreatic ductal adenocarcinoma (PDAC) is a rapidly increasing cause of mortality whose dismal prognosis is mainly due to overwhelming chemoresistance. New therapeutic approaches include physical agents such as ultrasonic cavitation, but clinical applications require further insights in the mechanisms of cytotoxicity. 3-D in vitro culture models such as spheroids exploit realistic spatial, biochemical and cellular heterogeneity that may bridge some of the experimental gap between conventional in vitro and in vivo experiments.

**Purpose:**

To assess the feasibility and efficiency of inertial cavitation associated or not with chemotherapy, in a spheroid model of PDAC.

**Methods:**

We used DT66066 cells, derived from a genetically-engineered murine PDAC, isolated from KPC-transgenic mice (LSL-Kras^G12D/+;^ LSL-Trp53^R172H/+;^ Pdx-1- Cre). Spheroids were obtained by either a standard centrifugation-based method, or by using a magnetic nano-shuttle method allowing the formation of spheroids within 24 hours and facilitating their handling. The spheroids were exposed to ultrasonic inertial cavitation in a specially designed setup. Eight or nine spheroids were analyzed for each of 4 conditions: control, gemcitabine alone, US cavitation alone, US cavitation + gemcitabine. Five US inertial cavitation indexes, corresponding to increased US intensities, were evaluated. The effectiveness of treatment was assessed after 24 hours with the following criteria: spheroid size (growth), ratio of phase S-entered cells (proliferation), proportion of cells in apoptosis or necrosis (mortality). These parameters were assessed by quantitative immunofluorescence techniques.

**Results:**

The 3D culture model presented excellent reproducibility. Cavitation induced a significant decrease in the size of spheroids, an effect significantly correlated to an increasing cavitation index (p < 0.0001). The treatment induced cell death whose predominant mechanism was necrosis (p < 0.0001). There was a tendency to a synergistic effect of US cavitation and gemcitabine at 5μM concentration, however significant in only one of the cavitation indexes used (p = 0. 013).

**Conclusion:**

Ultrasonic inertial cavitation induced a significant reduction of tumor growth in a spheroid model of PDAC., with necrosis rather than apoptosis as a Cell dominant mechanism of cell death. More investigations are needed to understand the potential role of inertial cavitation in overcoming chemoresistance.

## Introduction

Pancreatic ductal adenocarcinoma (PDAC), a currently leading cause of cancer mortality, has a rapidly increasing incidence and is expected to become the second cause of cancer death within 10–15 years [1]. The overall 5-year survival rate of PDAC does not exceed 8% [2]. Only 20% of patients have a resectable disease at diagnosis, and the 5-year survival rate in this surgical subgroup does not exceed 20%, as a result of early local and metastatic tumour spread as well as chemoresistance [3]. Even recent innovative combined chemotherapy regimens like Nab-pacitaxel + Gemcitabine [4], and FOLFIRINOX [5] have only marginally improved the median overall survival of non resectable pancreatic cancer and the surgical resection rates after neoadjuvant chemotherapy or chemo-radiation [6]. A major limitation in effectively treating PDAC using conventional and targeted chemotherapeutic agents is inadequate drug delivery to the target location, predominantly due to a poorly vascularized, desmoplastic tumor microenvironment, whose mass is predominant in PDAC [7].

Therapeutic focused ultrasound has been shown to have its own anti-tumoral effect, and also to improve drug delivery, in both *in vitro* and *in vivo* models of different types of [8–13]. Ultrasound can induce tumor cell death by focal heating (HIFU) or by the initiation, growth, oscillation and collapse of gas bubbles (cavitation). Cavitation creates tensile acoustic pressures larger than cohesion forces between molecules and thus enhances the penetration of drugs (steady cavitation) or creates irreversible damage to membranes and extracellular matrix (inertial cavitation). The physical properties of inertial cavitation make it likely to bear the most promising potential to overcome the barriers of PDAC microenvironment because disrupting the microenvironment in which PDAC cells are encased is considered a prerequisite to allow chemotherapy to penetrate membranes and induce tumor cell death. However, although some interesting results have been obtained by our group as well as others [13–15], and *Camus M*. *et al*, *COCHIN Institute*, *submitted*, ] small animal experiments have neither helped understand the mechanisms of cell death under ultrasonic cavitation nor optimize the combination of ultrasound and chemotherapy, a reason among others being that animal models do not offer sufficient reproducibility in terms of growth and response to draw robust conclusions.

To establish a tissue culture model simulating complex three-dimensional intercellular interactions, we worked on a model of multicellular spheroids of genetically engineered murine PDAC, including a three-dimensional (3D) cell culturing system based on magnetic bioprinting [16–17]. This model can help apprehend the effects of cavitation on cells and extracellular components of the microenvironment. In this first study, we aimed to evaluate the effects sustained by PDAC spheroids after exposure to inertial cavitation in the presence or absence of chemotherapy.

## Material and methods

### Cell culture

DT66066 cells were isolated from KPC-transgenic mice (K-ras^LSL.G12D/+^; p53^R172H/+^; PdxCre) [18–19]. DT66066 cells were cultured in Dulbecco's Modified Eagle's Medium (DMEM, Thermo Fischer Scientific, USA). Media were supplemented with 10% Fetal Bovine Serum (Thermo Fischer Scientific, USA), 1% penicillin G-streptomycin (Thermo Fischer Scientific, USA) and 1% ciprofloxacin (Ciflox, Bayer), and replaced every 3 days. Cells were cultured at 37°C with 5% CO_2_. At 80% confluency, the cells were detached with 0.25% trypsin for subculturing.

### “Nano-shuttle spheroids” by magnetic cell bioprinting

Three-dimensional bioprinting cell cultures were based on previously established methodology [16] and were set up using the 24-well Bio-Assembler kit (Nano3D Biosciences, Inc.) consisting of nano-shuttle (NS) solution and a 24-well plate magnetic drive. The NS is a nanoparticle assembly of iron oxide (Fe_2_O_3_) and gold (Au) nanoparticles cross-linked with poly-l-lysine to promote cellular uptake. The 24-well plates used for 3D culture were flat bottom ultra-low attachment plates.

At 80% confluency in a cell culture flask (25cm^2^, 75cm^3^, Thermo Fischer Scientific, USA) cells were mixed with 8μL of NS per cm2 plate area and placed in a standard CO_2_ cell culture incubator (37°C, 5% CO_2_ in air) for 12 hours in standard adherent culture conditions. Cells were then trypsinized to detach adherent cells from plate and to obtain single-cell suspension. After trypsin inactivation with serum, cells were counted, centrifuged, and seeded into a multiwell ultralow-attachment plate. The medium volume in each well was 0.4ml (cell concentration: 2.5 X 10^5^ cell per mL) for the 24-well plate. A magnetic drive was immediately placed under the culture to magnetically bioprint the cells and guide them to aggregate within hours. The cells aggregated just above each magnet, at the center of the well, where they self-assembled into a spheroid. Bioprinted spheroids were incubated in a CO_2_ incubator, and the magnetic drive was removed after 24 hours of incubation.

Because 3D cultures are magnetized, the same magnet drive was used to facilitate medium exchange throughout the 3D cell culturing process, and spheroid handling was achieved with a magnetic pen.

### Validation of nano-shuttle biocompatibility

Nano-shuttle is a nanoparticle assembly (~ 50 nm) consisting of gold, iron oxide, and poly-L-lysine (PLL), that attaches to the plasma membrane electrostatically. NS are then incorporated into cell cytoplasm, and released off the cell into the surrounding extracellular matrix over 16 hours for 2D cultures, and over 8 days for 3D cultures, [16–17].

Several publications have used 3D culturing cells based on NS with different cell lines, [14,17–23] and confirmed its biocompatibility. In particular these publications demonstrated that NS did not affect cell proliferation [20,21], viability [21], metabolism [20,22], inflammatory stress [20], or phenotype [16,20,22]. decided to investigate NS biocompatibility using DT66066 cells, and to evaluate the potential effect of these NS on chemotherapy and ultrasound cavitation cell sensitivity.

A transmission electron microscopy was performed to assess any cell architectural abnormality after 12 hours of incubation with NS. Cell proliferation, oxidative stress, gemcitabine cell sensitivity, and ultrasound cell sensitivity were studied with and without cell incubation with NS (12 hours of incubation).

These experiments were performed using samples of DT66066 cell suspension (for proliferation and chemosensitivity after incubation with NS: cell concentration was 10^4^ cell/ml in 200 mL of media per well for each sample; for the NS and cell sensitivity to US experiment, (including also NS and oxidative stress experiment) we used 10^5^ cells/ml in 600mL of media in a 2ml Eppendorf sterile tube for each sample).

### Ultrasound cavitation and chemotherapy treatments

The ultrasonic setup used has been previously described by Chettab et al. [23], but adjusted in our study to generate inertial cavitation instead of stable cavitation.

Briefly, it is composed of two piezo-ceramic focused transducers of 50 mm diameter, 50mm curvature, and F = 1.1 MHz frequency. These are separated by an angle of 90° and placed in order to match their respective focal points. An in-house hydrophone is placed in the water tank to record acoustic cavitation emissions from the focal area. The excitatory signal is generated by a waveform generator (PXI 5412 National Instruments, Austin,TX) and amplified with a 200 W power amplifier (LA200H, Kalmus, Bothell, WA). This signal is a burst of 2750 sinusoidal cycles with frequency of 1.1 MHz and a repetition frequency of 100 Hz, mostly generating an inertial cavitation regimen. The setup was filled with Ablasonic (EDAP-TMS, France), a cavitation-inhibiting fluid, preventing acoustic cavitation occurrence outside the sample submitted to ultrasound.

Each spheroid was individually placed in an 2ml sterile Eppendorf tube (Eppendorf, Germany), and placed in the center of the setup, so that the spheroid was inevitably in the focal point. Several inertial cavitation index (CI) were tested (CI 10, CI 14, CI 18, CI 22 et CI 26, during 20 seconds), corresponding to crescent US intensities.

A pre-treatment with gemcitabine at a concentration of 5 μM was performed during 4 h before the US application in designated spheroid groups. The other groups of spheroids included three conditions: no-treatment, US treatment only, gemcitabine treatment only. Spheroids were then replaced in their initial media (i.e. with or without gemcitabine) and incubated during 24 hours.

### Evaluation of cell proliferation, and oxidative stress

The UptiBlue Viable Cell Counting assay was used to measure quantitatively the proliferation.

Intra-cellular oxidative stress was evaluated by glutathione and H2O2 measurement using a spectrofluorimeter (Fusion microplate reader fluorometer, Packard).

Cell viability, glutathione and H2O2 measurement were calculated using the GraphPad Prism 5.0 software (GraphPad Software Inc., San Diego, CA) was expressed relative to untreated control.

### Immunofluorescence evaluation of US cavitation +/- chemotherapy treatment efficiency

Three end-points were evaluated: proliferation, apoptosis and necrosis.

Cell proliferation was quantified using the Click-it EdU Assay (Alexa fluor 488, Invitrogen, Life Technologies, USA). This assay is an alternative to the BrdU assay. EdU (5-ethynyl-2´-deoxyuridine) provided in the kit is an analog of thymidine and is incorporated into DNA during active DNA synthesis. A concomitant labeling of spheroids by DAPI (DAPI nucleic acid stain, Invitrogen, Life Technologies, USA) fluorescence was performed.

Apoptosis and necrosis were quantified using respectively YO-PRO-1 (YO-PRO-1 (491/509), Invitrogen, Life Technologies, USA) and Propidium iodide (Propidium Iodide (535/617), Invitrogen, Life Technologies, USA) labeling.

### Images acquisition and analysis

Transmission electron microscopy (TEM) was performed with a Jeol transmission electron microscope (Jeol TEM, USA).

Fluorescence images from spheroids were acquired using a straight confocal microscope, SPINNING DISK Leica DM6000 (Leica, Germany), using a 25X objective. Images were processed using Metamorph (Molecular Devices, USA) and ImageJ (ImageJ software, W.Rasband, NIH, USA) softwares.

The size of spheroids was estimated by measuring the largest diameter. Measuring Cell Fluorescence using ImageJ, was fulfilled by a method previously described [24,25] based on the following formula:

Corrected Total Cell Fluorescence (CTCF) = Integrated Density–(Area of selected cell X Mean fluorescence of background readings)

### Statistical analysis

Data are presented as mean ± standard deviation (SD). An ANOVA test was performed considering the number of conditions studied (more than 2). Mann-Whitney test was used for pharmacokinetic results (i.e validation of nanoparticles biocompatility). Statistical analyses were performed by Graphpad Prism software (GraphPad Software, Inc., La Jolla, California, USA). Results were considered to be statistically significant for a p value <0.05.

## Results

### Validation of nano-shuttle (NS) biocompatibility

**Fig 1:**
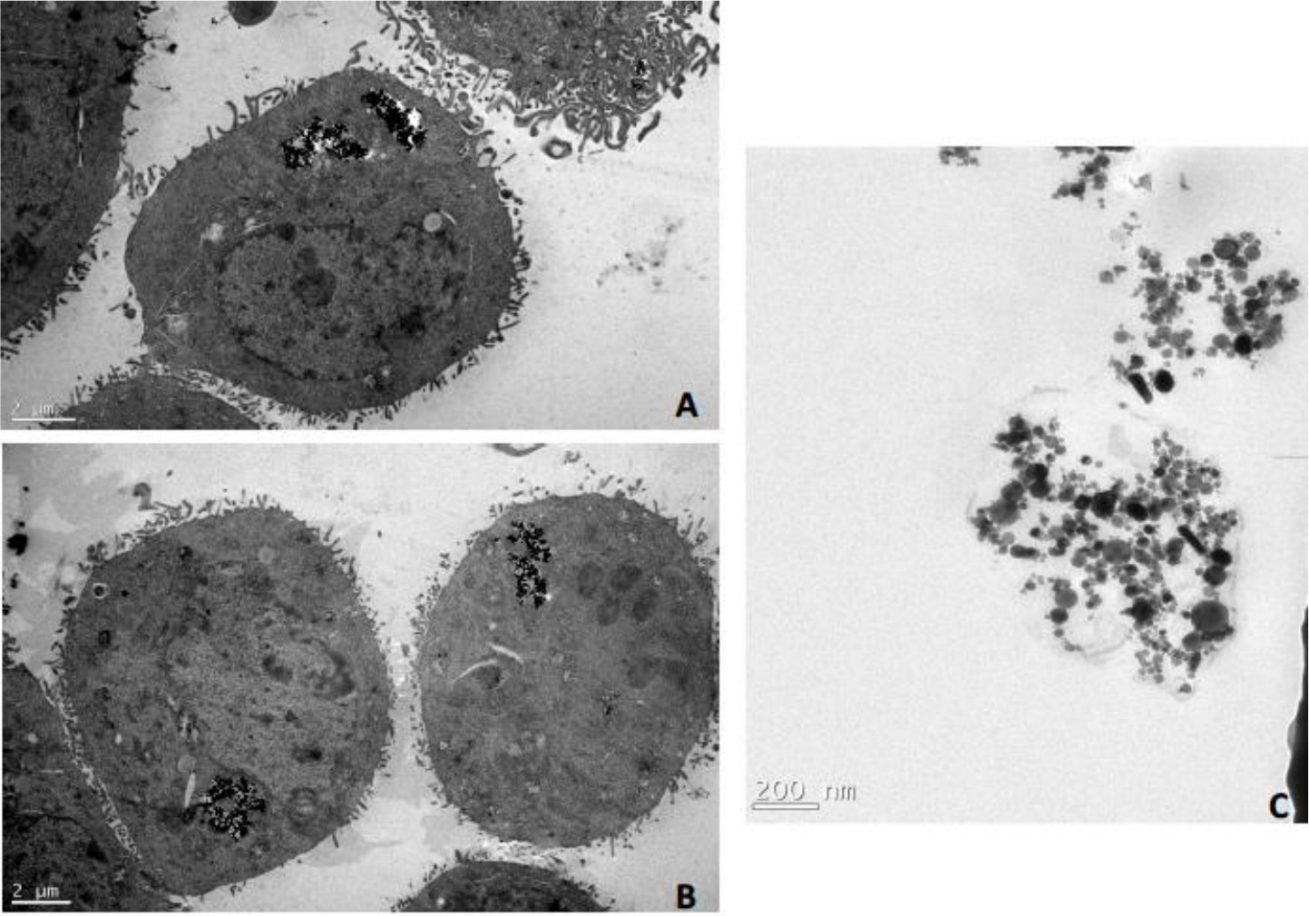
Transmission electron microscopy acquisition of DT66066 cells after 12 hours of incubation with NS. **(A)** and (**B)**: NS are incorporated into cell cytoplasm inside endocytic vacuoles; **C**: NS are released off the cell over 7–8 days into the surrounding extracellular matrix.

Fig 1 shows a transmission electron microscopy acquisition of DT66066 cells after 12 hours of incubation with NS. NS are incorporated into cell cytoplasm inside endocytic vacuoles. No NS were detected into cell nuclei, and no architectural abnormalities were identified.

**Fig 2:**
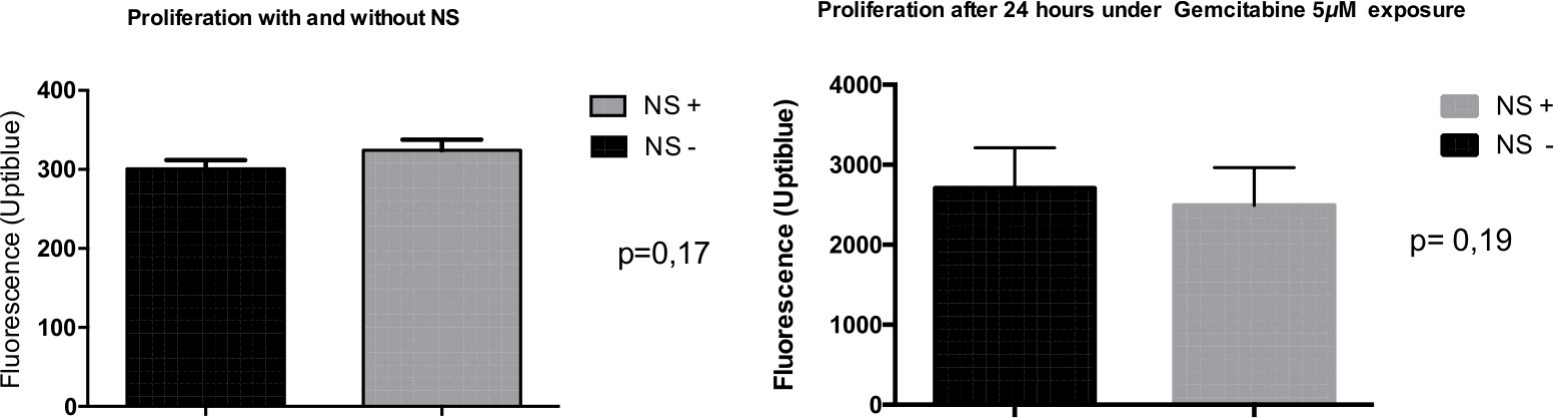
Cell proliferation and chemosensitivity to gemcitabine after incubation with NS (17–20 samples for each condition). Statistical analysis performed with Mann-Whitney test. NS impacted neither cell viability, proliferation, or chemosensitivity under gemcitabine exposure. *NS +*: *DT66066 cells incubated with NS; NS—*: *DT66066 cells without incubation with NS*.

For each condition, 17–20 samples were analyzed. NS affected neither cell viability, proliferation, or chemosensitivity under gemcitabine exposure (Fig 2).

**Fig 3:**
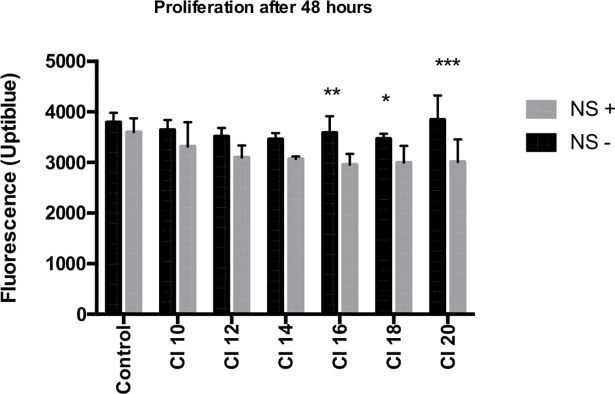
NS and cell sensitivity to US. 6 samples for each condition (a total of 84 samples). Statistical analysis performed with ANOVA test. *NS +*: *DT66066 cells incubated with NS; NS—*: *DT66066 cells without incubation with NS*.

For each condition, 6 samples were analyzed (total 84 samples). NS potentiated ultrasound cell sensitivity at highest levels of cavitation index—CI 16, CI18 and CI 20 (respectively p = 0,0024, p = 0,0417, p<0,0001)—as shown by a reduction of cell proliferation for the DT66066 cells incubated with NS (NS+) (Fig 3), but not at lower CI indexes.

**Fig 4:**
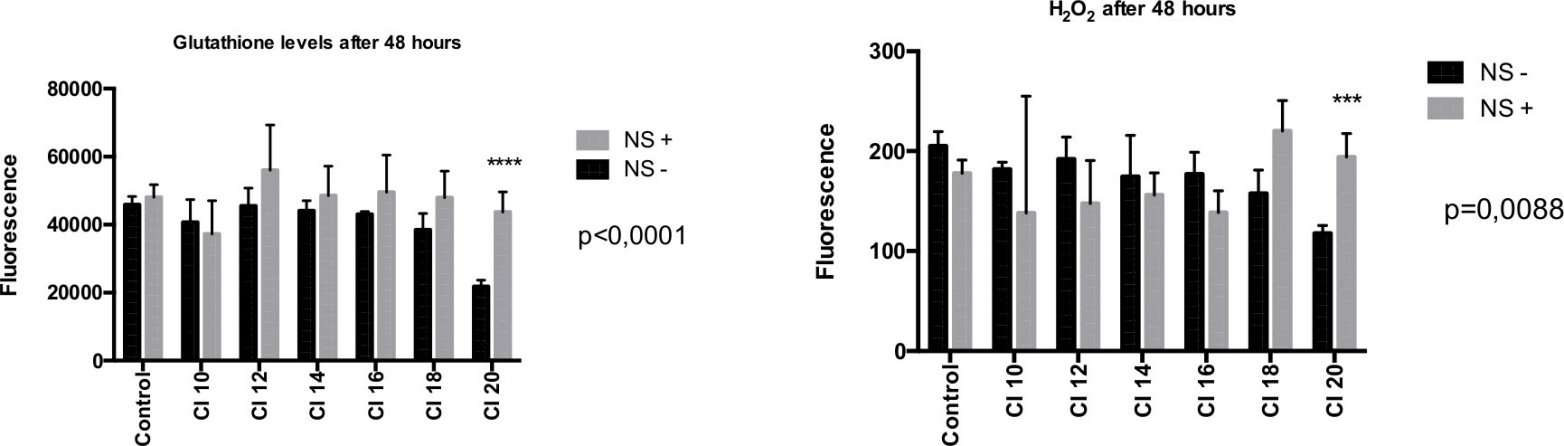
NS and oxidative stress. 6 samples for each condition (total of 84 samples). Statistical analyze performed with ANOVA test. *NS +*: *DT66066 cells incubated with NS; NS—*: *DT66066 cells without incubation with NS*.

For each condition 6 samples were analyzed (a total of 84 samples for glutathione experiments and 84 other samples for H_2_O_2_ experiments). NS did not affect oxidative stress for most cavitation indexes. Glutathione and H_2_O_2_ levels were only significantly elevated for NS+ cells when treated with the highest cavitation index tested, i.e. CI 20 (respectively p<0,0001, p = 0,0088) (Fig 4).

### US cavitation treatment

At 72 hours of growth, spheroids were treated with increasing US inertial cavitation index, preceded or not by a 4 hours 5 μM gemcitabine incubation.

**Fig 5:**
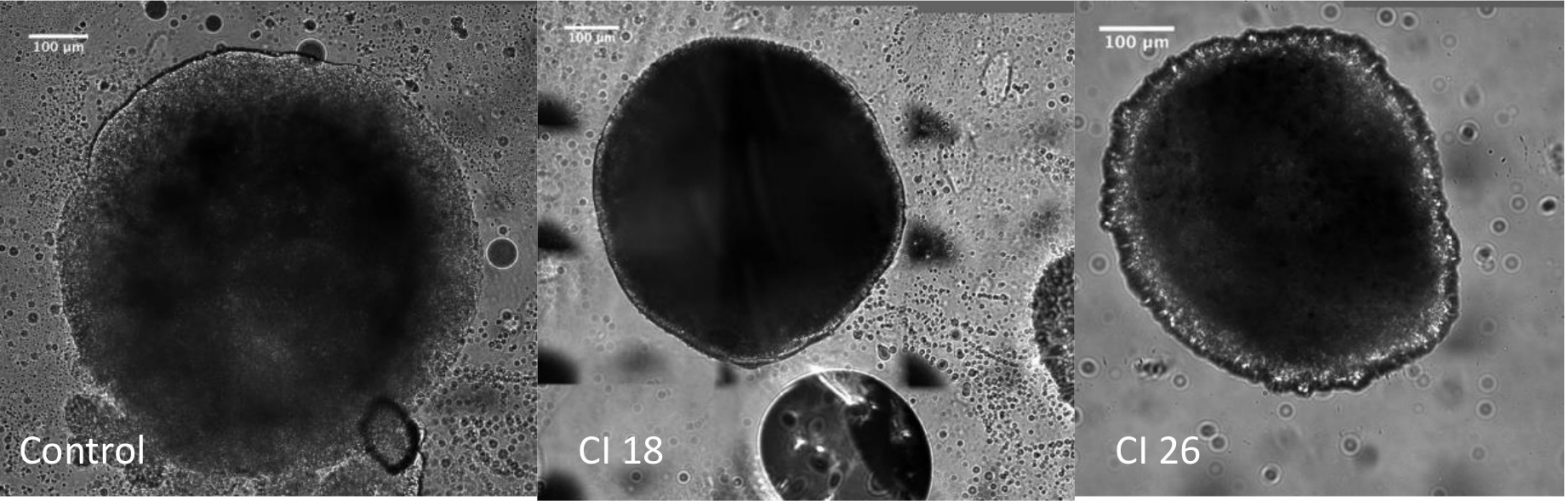
Examples of spheroids after inertial cavitation treatment. Images acquired using a straight confocal microscope, SPINNING DISK Leica DM6000 (Leica, Germany), 25X objective. *CI*: *Cavitation Index*.

**Fig 6:**
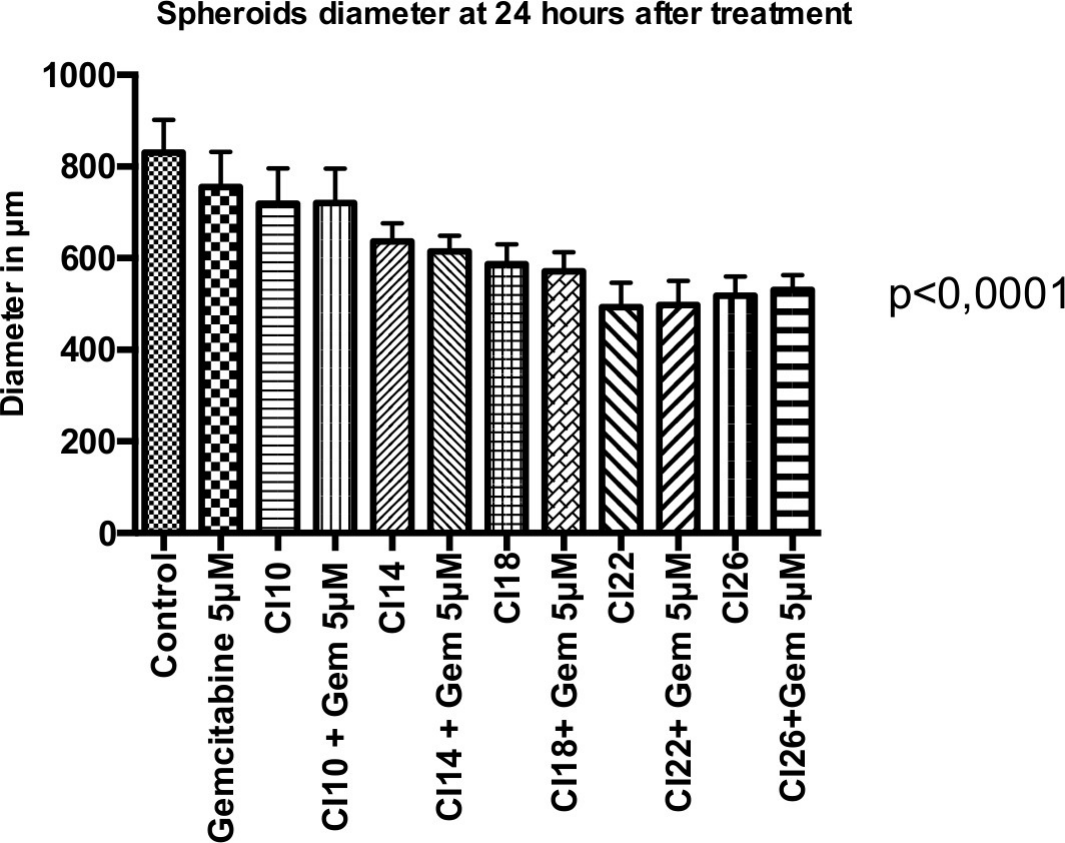
Size of spheroids at 24 hours after treatment. 14–18 spheroids for each condition. Statistical analysis performed with ANOVA test. *CI*: *Cavitation Index; Gem 5μM*: *Gemcitabine at a concentration of 5 μM*.

For each condition, 14 to 18 spheroids were analyzed. The median diameter of non-treated spheroids (control) was 830.80 μm (SD = 70.89 μm). The average thickness was 300 to 350 μm. There was a significant and progressive reduction of spheroid diameter correlated with the increase of the cavitation index (Fig 5, Table 1, and Fig 6) (p< 0.0001).

**Table 1:** Size of spheroids at 24 hours after treatment.

	Control	Gem5 μM	CI10	CI10 + Gem5 μM	CI14	CI14 + Gem5 μM	CI18	CI18 + Gem5 μM	CI22	CI22 + Gem5 μM	CI26	CI26 + Gem5 μM
**Diameter** **(mean in μm)**	830.80	755.50	719.00	720.10	636.10	614.40	587.00	571.30	492.80	498.00	517.60	530.70
**Standard deviation (m)**	70.89	76.76	77.64	75.68	40.49	34.63	43.03	41.37	53.35	52.14	42.39	32.42
**p value**		0,02	<0.0001	<0.0001	<0.0001	<0.0001	<0.0001	<0.0001	<0.0001	<0.0001	<0.0001	<0.0001

14–18 spheroids for each condition. Statistical analysis performed with ANOVA.

CI: Cavitation Index; Gem 5 μM: Gemcitabine at a concentration of 5 μM

**Fig 7:**
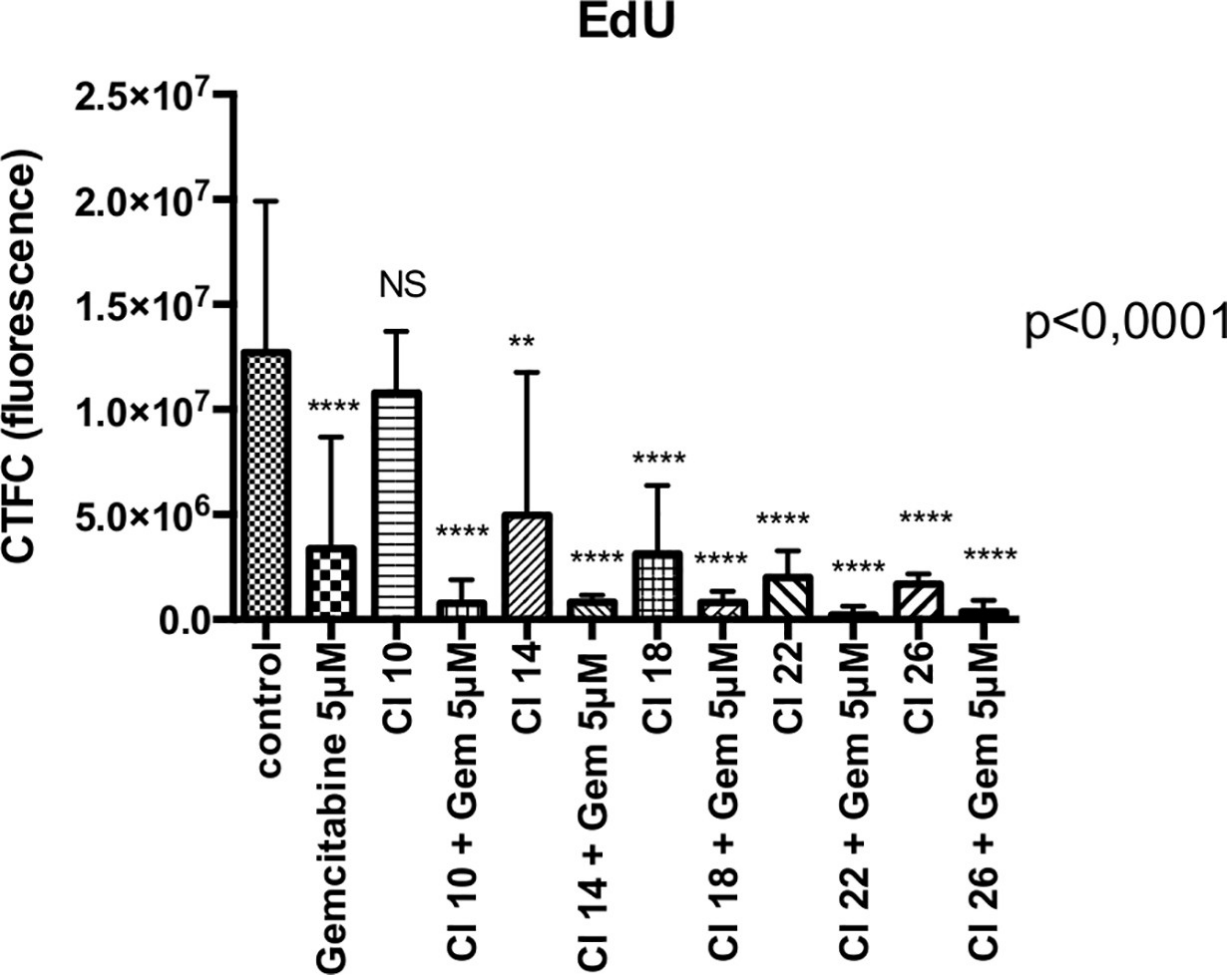
Cell proliferation quantified by EdU fluorescence. 8 or 9 spheroids per condition. Statistical analysis performed with ANOVA. *CI*: *Cavitation Index; Gem 5 μM*: *Gemcitabine at a concentration of 5 μM*

**Fig 8:**
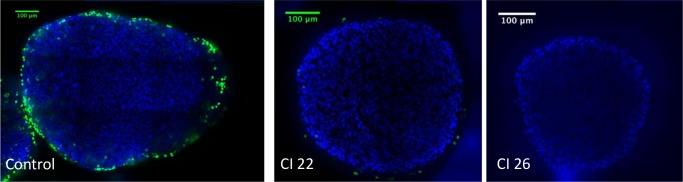
Examples of EdU plus DAPI fluorescence after inertial cavitation treatment. DAPI stains cell nuclei with a blue fluorescence. EdU stains phase S–entered cells with a green fluorescence. Images acquired using a straight confocal microscope, SPINNING DISK Leica DM6000 (Leica, Germany), 25X objective. *CI*: *Cavitation Index*.

EdU fluorescence quantification showed a significant reduction in cell proliferation with US intensity increment and with gemcitabine association (n = 8 or 9 spheroids per condition, p<0.0001) (Fig 7 and Fig 8), which was observed with all CI except CI 10 (p = 0.99).

**Fig 9:**
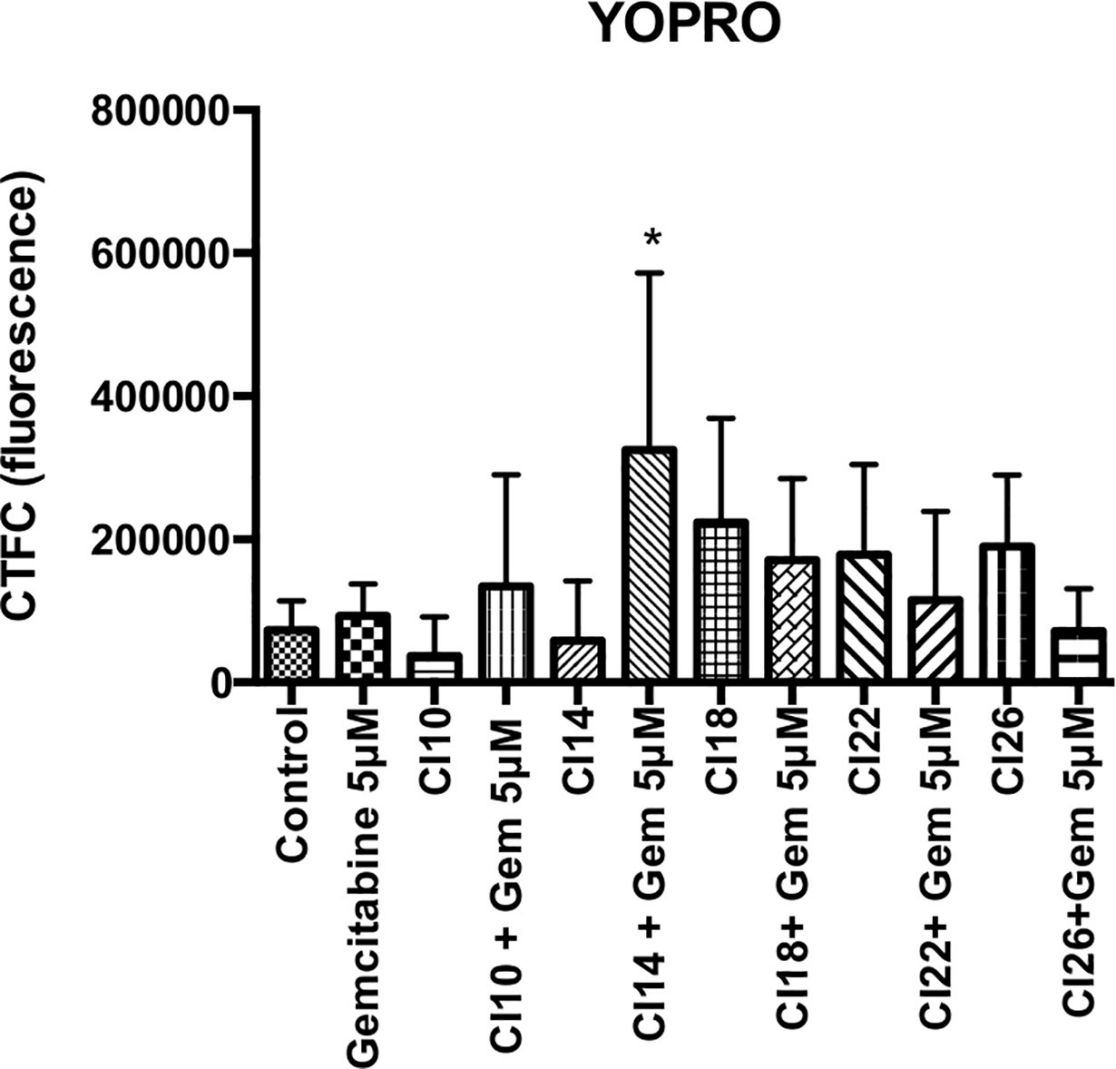
Cell apoptosis quantified by YO-PRO-1 fluorescence (without Propidium iodide fluorescence). 8 or 9 spheroids per condition. Statistical analysis performed with ANOVA. *CI*: *Cavitation Index; Gem 5 μM*: *Gemcitabine at a concentration of 5 μM*.

**Fig 10:**
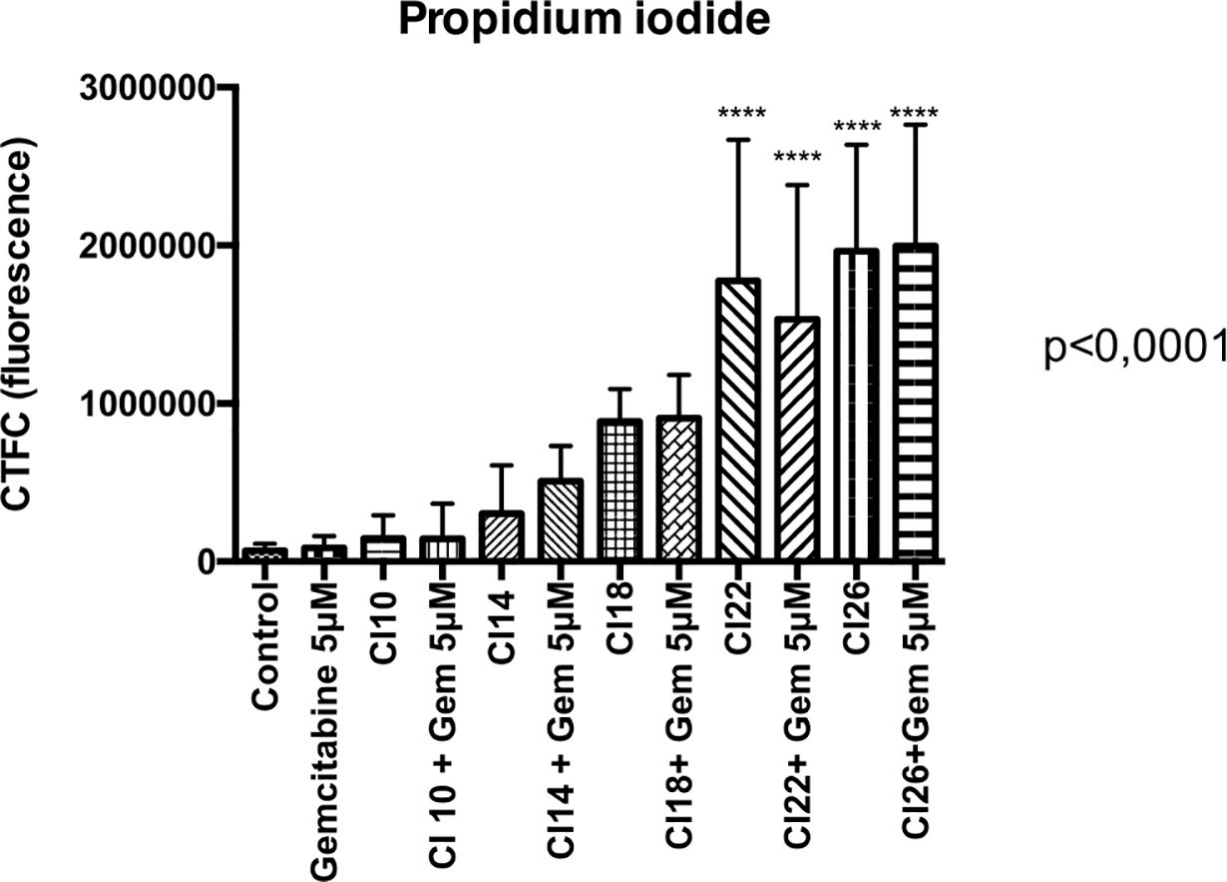
Propidium iodide fluorescence. 8 or 9 spheroids per condition. Statistical analysis performed with ANOVA. *CI*: *Cavitation Index; Gem 5 μM*: *Gemcitabine at a concentration of 5 μM*.

**Fig 11:**
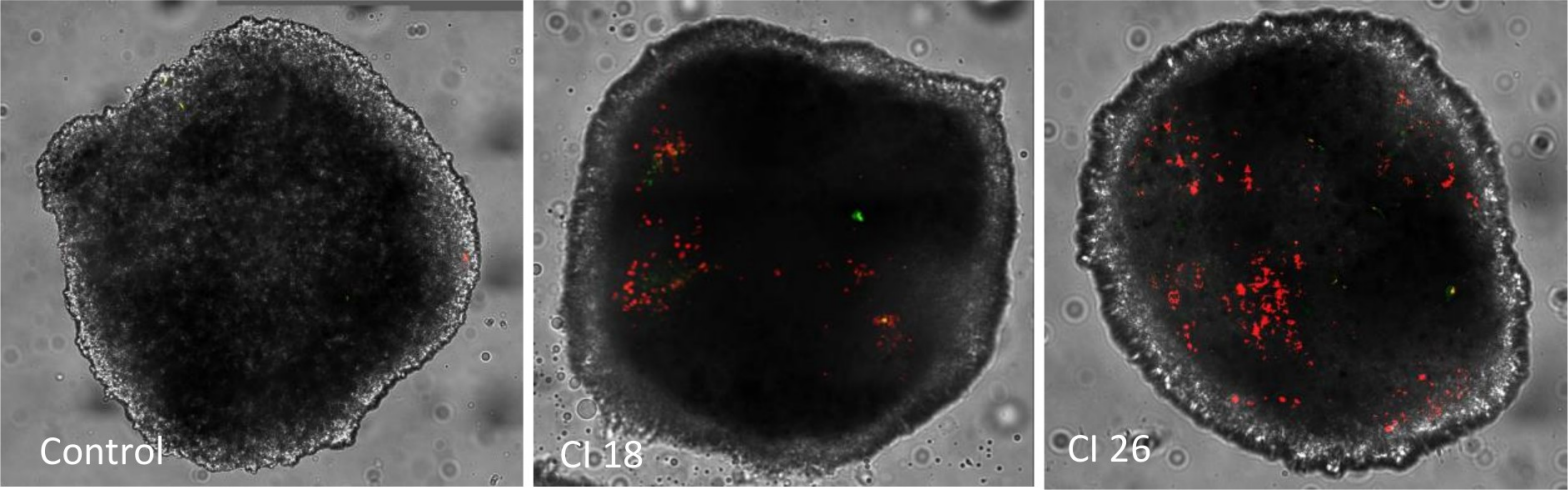
Examples of YO-PRO-1 fluorescence (green fluorescence) and Propidium iodide fluorescence (red fluorescence). Images acquired using a straight confocal microscope, SPINNING DISK Leica DM6000 (Leica, Germany), 25X objective. *CI*: *Cavitation Index*.

Cells death was predominantly mediated by a necrosis mechanism, increased with US intensity increment as shown by Fig 9 and Fig 10 (n = 8 or 9 spheroids per condition, p<0.0001). There was a weak apoptotic effect, only significant for the condition “CI 14 + gemcitabine”(Fig 11).

## Discussion

Although the cytotoxicity of ultrasound, including inertial cavitation, has been described in various preclinical models for several decades [8,9], efforts to translate these effects in animal models have been sometimes disappointing [14,15]. This may result from an insufficient understanding of the optimal ultrasound regimen to be applied *in vivo* when the objective is either to kill cancer cells by mechanical means (while sparing healthy tissue) or to increase the penetration of drugs into malignant cells to potentiate chemotherapy. This is all the more important in the case of PDAC, where chemoresistance is especially overwhelming and the microenvironment is a particularly active player in the defense and promotion of cancer growth. We considered a good way to overcome some of these hurdles would be to proceed in a stepwise fashion, starting with a 3D model of PDAC. Three-dimensional cell culture models provide a more representative model of tumor growth and chemotherapy response than usual 2D models, because 3D models display spatial arrangement, biochemical and cellular heterogeneity that are more representative of *in vivo* tumor behavior. We started with a conventional, centrifugation-based spheroid model, then moved to a model based on magnetic nano-shuttle. To our knowledge, this work is the first to apply this method of 3D culture engineering to murine pancreatic adenocarcinoma cells. This 3D-nanoshuttle model provides excellent reproducibility and an easier handling of spheroids than a previous model we used, cultured in a more conventional manner [26–28]. This “conventional spheroid model”, permitted to demonstrate some effects on tumor growth and synergism with chemotherapy on CAPAN-2-derived spheroids, but slower spheroid formation and more difficult handling for back and forth transfers between culture pits and ultrasonic exposures, which made it more difficult to analyze proliferation, necrosis and apoptosis [26]. A possible limitation of this work is the effect of the nanoparticles used to generate the 3D model. Indeed, although there was no impact on cell viability, proliferation, gemcitabine cell sensitivity, or oxidative stress, there was a significant reduction of cell proliferation for the highest cavitation index tested. As previously described, nanoparticles are eliminated from the spheroid at the eighth day of growth [16]. However, similar findings with both models in terms of growth impairment is a strong argument for their validity and against any bias induced by the magnetic nanoparticles in the nano-shuttle model. Our results reveal a therapeutic effect of inertial cavitation when combined with gemcitabine, but also when used alone. A decrease in size of the spheroids was the first significant observation, which was well correlated with increasing ultrasound intensity/index. In the same way, US treatment induced cell death whose predominant mechanism was necrosis. Different biophysical properties associated with inertial cavitation may explain these results: an increased cell membrane permeability, a modification of ionic homeostasis, the generation of reactive oxygen species (ROS), as well as ultrasound-specific thermal effects, any or a combination of which can lead to cell death [29–32].

Inertial cavitation also conferred a synergistic effect when combined with chemotherapy, which can be explained by several mechanisms: an increased cellular chemosensitivity, a modification of the drug’s molecular structure leading to a more active or a more cytotoxic form, and an increased intra-cellular concentration of the chemotherapy. We observed a synergistic effect of inertial cavitation combined with gemcitabine at a concentration of 5 μM, but this was significant only for a cavitation index CI14, which is probably due to the fact that Gemcitabine is already cytotoxic at this concentration, thus explaining the limited synergism of the combination US + gemcitabine. Previous experiments carried out at a concentration of 20 μM showing a significant cytotoxicity on DT66066 cells in 2D cultures prompted us to reduce the concentration to 5 μM. Our results suggest that further reduction in the concentration of gemcitabine, in combination with inertial cavitation, would possibly exhibit more potent synergistic effects.

In this first step of the study of PDAC spheroids and inertial cavitation, the microenvironment tumor components were not included, although they are known to play a major role in the chemoresistance of pancreatic adenocarcinoma [33,34]. This is indeed an important limitation of this study. The next steps of this project will include the development of 3D co-culture models of pancreatic adenocarcinoma that incorporate the tumor microenvironment, and applying US treatment after eight days of spheroid growth in order to eliminate residual nanoparticles. Another important player in tumor development and drug distribution is vascularity, which is absent in spheroid models. Vascularized models may thus be necessary at a later stage, in the form of either organoids or genetically engineered animal models. However, the spheroid model appears to be particularly suitable for the reliable and reproducible analysis of short term cavitation-induced mechanical effects.

In conclusion, ultrasonic inertial cavitation applied in a 3D model of genetically engineered murine PDAC cell culture allowed a significant reduction of tumor growth, induced cells necrosis and synergized Gemcitabine under certain cavitation conditions. More investigations involving the development of a model including tumor microenvironment cells are needed in the translational process towards a clinical application of ultrasonic cavitation to PDAC therapy.

## Abbreviations

3D: three dimensional
CI: Cavitation Index
Gem 5 μM: Gemcitabine at a concentration of 5 μM
NS: nano shuttle
NS+ cell: cell incubated with NS
US: ultra sound
